# A mix-and-click method to measure amyloid-β concentration with sub-micromolar sensitivity

**DOI:** 10.1098/rsos.170325

**Published:** 2017-08-09

**Authors:** Christine Xue, Yoon Kyung Lee, Joyce Tran, Dennis Chang, Zhefeng Guo

**Affiliations:** Department of Neurology, Brain Research Institute, Molecular Biology Institute, University of California, Los Angeles, CA 90095, USA

**Keywords:** fluorescamine, Alzheimer's disease, amyloid, Aβ40, Aβ42, protein aggregation

## Abstract

Aggregation of amyloid-β (Aβ) protein plays a central role in Alzheimer's disease. Because protein aggregation is a concentration-dependent process, rigorous investigations require accurate concentration measurements. Owing to the high aggregation propensity of Aβ protein, working solutions of Aβ are typically in the low micromolar range. Therefore, an ideal Aβ quantification method requires high sensitivity without sacrificing speed and accuracy. Absorbance at 280 nm is frequently used to measure Aβ concentration, but the sensitivity is low with only one tyrosine and no tryptophan residues in the Aβ sequence. Here we present a fluorescence method for Aβ quantification using fluorescamine, which gives high fluorescence upon reaction with primary amines. We show that, using hen egg white lysozyme as a standard, fluorescence correlates linearly with primary amine concentration across a wide range of fluorescamine concentrations, from 62.5 to 1000 µM. The maximal sensitivity of detection is achieved at a fluorescamine concentration of 250 µM or higher. The fluorescamine method is compatible with the presence of dimethyl sulfoxide, which is commonly used in the preparation of Aβ oligomers, and limits the use of absorbance at 280 nm due to its high background reading. Using aggregation kinetics, we show that the fluorescamine method gives accurate concentration measurements at low micromolar range and leads to highly consistent aggregation data. We recommend the fluorescamine assay to be used for routine and on-the-fly concentration determination in Aβ oligomerization and fibrillization experiments.

## 1. Introduction

The aggregation of amyloid-β (Aβ) protein leads to the formation of a wide range of aggregates, from soluble oligomers to insoluble fibrils [1]. These Aβ aggregates play a central role in the pathogenesis of Alzheimer's disease [2]. Because protein aggregation is a concentration-dependent process, quantitative analysis of Aβ aggregation and rigorous studies of the bioactivity of these Aβ aggregates would require accurate measurement of Aβ concentration. Two characteristics of the Aβ protein restrict the use of otherwise commonly employed protein quantification methods. The first characteristic is its high aggregation propensity. Aβ42 aggregation typically occurs within hours at 37°C [3,4]. This leads to the second characteristic of Aβ protein, that working solutions of Aβ are typically in the low micromolar range. In order to acquire the concentration of an Aβ42 sample in its pre-fibrillar state, the quantification method must be fast and sensitive. A commonly used protein quantification method is absorbance at 280 nm. Because Aβ42 has only one tyrosine and no tryptophan residues, the extinction coefficient at 280 nm is very low, at 1280 M^−1^ cm^−1^ [5]. To achieve an absorbance reading of 0.1 at 280 nm, the concentration of Aβ needs to be approximately 80 µM. Working solutions of Aβ42 are rarely that high in concentration, and typical concentrations for Aβ42 aggregation experiments (less than 50 µM) fall well below the sensitive range of UV absorbance [6,7]. Use of dimethyl sulfoxide (DMSO) as a solubilizing reagent for some Aβ protocols (e.g. Aβ-derived diffusible ligands, or ADDLs) [8] further limits the use of absorbance due to the high background signal of DMSO.

Likely due to the lack of fast and sensitive quantification methods for Aβ, many Aβ studies have relied on ‘nominal' concentrations without actually measuring them. For example, several studies [9–12] on ADDLs rely on the accurate quantity of starting Aβ material, often provided by the vendor, and no Aβ quantifications were performed to verify the concentration. Biochemical and biophysical characterization of these Aβ oligomers reveals large variations in their characteristics, from morphology to size. Freir *et al*. [11] reported ADDLs that are rich in elongated structures, while Laurén *et al.* [10] reported ADDLs that are dominantly globular. Studies by both Freir *et al*. [11] and Laurén *et al.* [10] show that ADDLs are large on the scale of hundreds of kilodaltons, but studies by the William Klein laboratory, the group that discovered these oligomers, show that active ADDLs have sizes of 10–100 kDa [13]. Accurate Aβ quantification may not completely resolve the inconsistencies from one laboratory to another, but it will certainly be a concrete first step to improve data reproducibility in Aβ research.

Here we propose to use a fluorescamine assay as a routine and on-the-fly method for Aβ quantification. Fluorescamine is a non-fluorescent compound that reacts with primary amines to produce highly fluorescent products [14]. Since its introduction by Udenfriend *et al*. [14] in 1972, several studies [15–20] have investigated the mechanism and optimization of fluorescamine assays. However, the fluorescamine method has never become a routine technique for protein quantification. Compared with other commonly used protein quantification methods such as Lowry [21], Bradford [22] and BCA [23], the major advantage of the fluorescamine method is that it does not require incubation and thus is truly a mix-and-click method. The only limitations of the fluorescamine method are that it relies on the knowledge of the number of accessible lysines in the protein and that the buffer cannot contain any primary amines (ruling out the use of glycine or Tris). In the particular case of Aβ, these conditions are usually satisfied as Aβ experiments are generally performed with purified Aβ in PBS buffer. Here, we show that fluorescamine is an ideal reagent for the quantification of low concentrations of Aβ proteins, in that the method is both fast and highly sensitive. We show that hen egg white lysozyme, with its primary amines readily accessible [24], is a suitable protein standard for the fluorescamine method. We explore a range of concentrations of fluorescamine for the quantification of Aβ, and finally, we show that highly consistent Aβ aggregation kinetics can be obtained when Aβ concentration is determined using the fluorescamine method.

## 2. Results and discussion

### 2.1. Choice of lysozyme as the protein standard for the fluorescamine method

Lysozyme is a commercially available protein standard. It is extremely stable, with a melting temperature of 74°C at neutral pH [25]. Most importantly, lysozyme contains seven primary amine groups, all of which can be readily modified [24]. This property makes lysozyme an ideal standard for protein quantification using fluorescamine, whose fluorescence depends on reaction with primary amine groups. By contrast, bovine serum albumin (BSA), a more commonly used standard for protein quantification, has 60 primary amine groups, and 20 of these amines are buried and cannot be readily labelled [26]. Variations in the extent of fluorescamine modification in albumin would lead to large measurement errors in protein quantification. Therefore, even though BSA has been used as a standard for fluorescamine assays in the literature [27], we consider it not an ideal standard for this particular application.

### 2.2. Standard curves and Aβ concentration measurements at different fluorescamine concentrations

**Figure 1.**
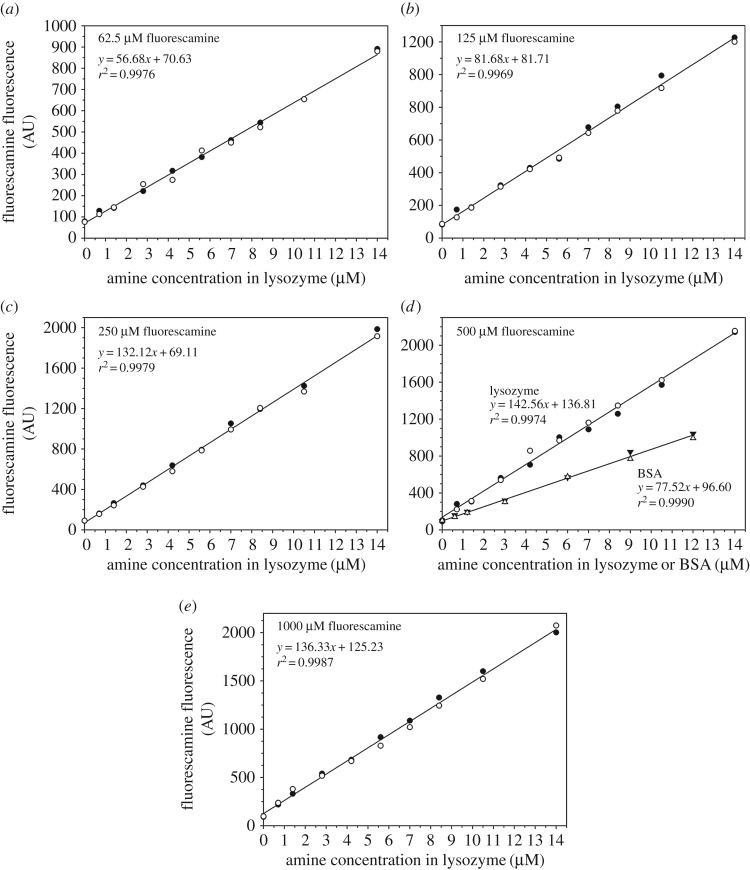
Standard curves for the fluorescamine method. Hen egg white lysozyme at nine concentrations (0.1, 0.2, 0.4, 0.6, 0.8, 1.0, 1.2, 1.5, 2.0 µM) was used to derive the standard curves. Concentrations of fluorescamine are at 62.5 µM (*a*), 125 µM (*b*), 250 µM (*c*), 500 µM (*d*) and 1000 µM (*e*). For comparison, BSA was also used for the 500 µM fluorescamine measurements (*d*). Two independent measurements were performed. Lines are linear fits to the data.

Previous fluorescamine methods of protein quantification have used a wide range of fluorescamine concentrations, including 90 µM [14], 120 µM [19], 216 µM [20], 250 µM [15,16], 360 µM [18], 1.6 mM [28] and 2.2 mM [27]. To examine the precision of protein quantification using various fluorescamine concentrations, and to see if there exists a fluorescamine concentration with the greatest sensitivity of detection, we tested five fluorescamine concentrations at 62.5, 125, 250, 500, 1000 µM and obtained standard curves using hen egg white lysozyme (figure 1). At all fluorescamine concentrations, a linear relationship was found for lysozyme concentrations in the range of 0.1–2 µM, corresponding to amine concentrations of 0.7–14 µM. For comparison, we obtained a BSA standard curve at 500 µM of fluorescamine (figure 1*d*). When plotted as a function of amine concentrations, the BSA standard gives a lower slope than the lysozyme data, due to the presence of buried lysines in BSA [26].

In all the standard curves with lysozyme, the lowest lysozyme concentration was 0.1 µM, and this corresponds to an amine concentration of 0.7 µM. Because Aβ consists of three primary amines, the 0.7 µM amine concentration can be translated into 0.23 µM Aβ concentration. Therefore, these standard curves suggest that the fluorescamine method may be able to detect Aβ at as low as 0.23 µM. The detection sensitivity of the fluorescamine method is further investigated below.

**Figure 2.**
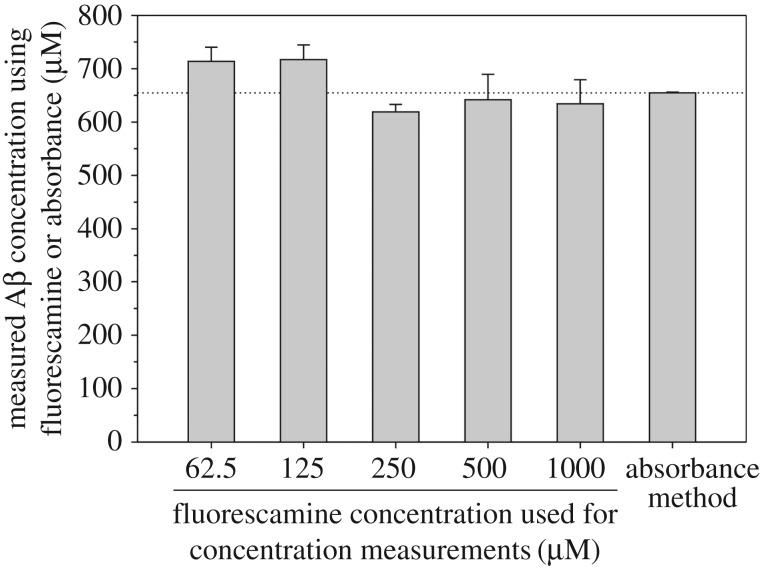
Comparison of Aβ40 concentrations determined using the fluorescamine method with absorbance at 280 nm. The dotted horizontal line is drawn to aid visual comparison. Error bars are standard deviations of three independent measurements. For the fluorescamine data, the error includes fluctuations in both dilution and instrument. For the absorbance data, the error bar reflects only instrument error and is thus extremely small as the sample was measured without dilution.

To establish a comparison between concentration measurements using fluorescamine and absorbance at 280 nm, we determined the concentration of Aβ40 using both methods (figure 2). For this purpose, we compared two tubes of Aβ40 powder with equal weight. One tube of Aβ40 was dissolved in a high pH denaturing buffer containing 20 mM CAPS (pH 11) and 7 M guanidine hydrochloride (GdnHCl). The absorbance at 280 nm was measured without further dilution to achieve a reading between 0.5 and 1.0. Then the concentration was calculated using an extinction coefficient of 1280 M^−1^ cm^−1^ [5]. For the fluorescamine method, the other tube of Aβ40 was dissolved in one volume of 10 mM NaOH, followed by addition of eight volumes of PBS and one volume of 10 mM HCl. The Aβ40 sample was diluted with PBS so that a fluorescence reading in the middle of the standard curve could be obtained, corresponding to an Aβ40 concentration of approximately 2 µM. Aβ40 concentration was determined using different fluorescamine concentrations (62.5, 125, 250, 500 and 1000 µM), and the results are shown in figure 2. In general, the measured Aβ40 concentrations using the five fluorescamine concentrations are in good agreement with the concentration determined using UV absorbance. For reasons we do not fully understand, the measured Aβ concentrations obtained using the low fluorescamine concentrations (62.5 and 125 µM) are higher than that obtained using absorbance, while the high fluorescamine concentrations (250, 500 and 1000 µM) yielded lower concentrations than the absorbance method (figure 2). It is also worth pointing out that the measured Aβ concentrations using high fluorescamine concentrations (greater than 250 µM) have better agreement with the absorbance method.

### 2.3. Fluorescamine method is compatible with high concentrations of dimethyl sulfoxide and hexafluoroisopropanol

**Figure 3.**
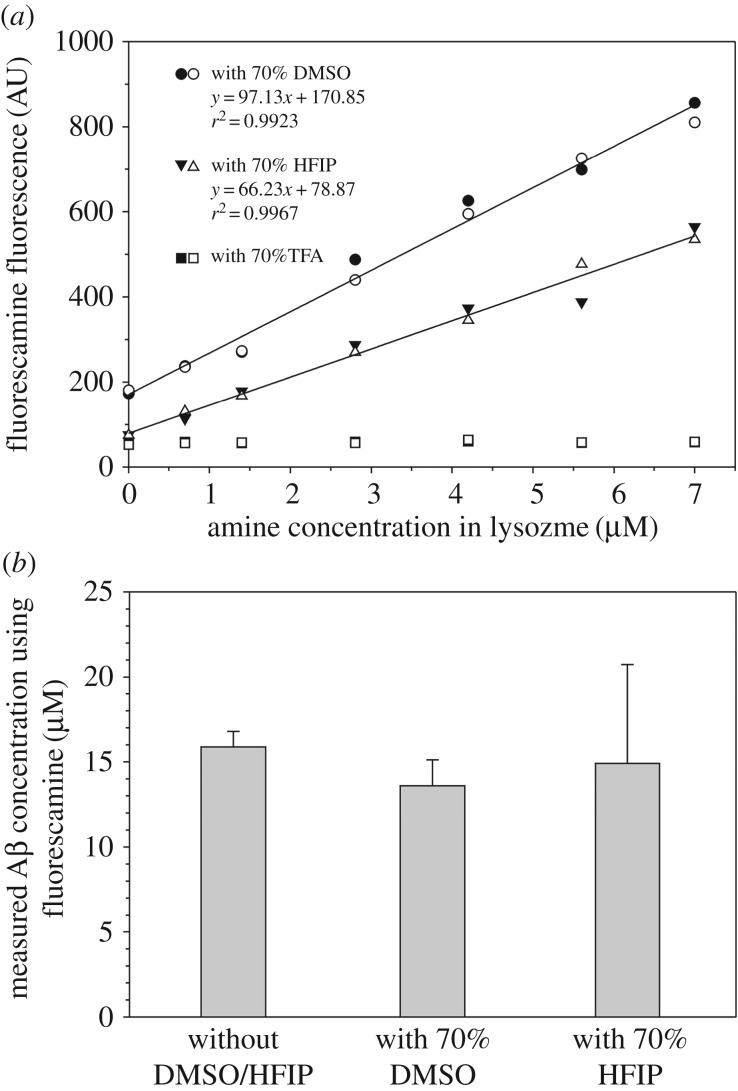
Fluorescamine method is compatible with DMSO and HFIP. (*a*) Standard curve using hen egg white lysozyme in the presence of 70% DMSO (open and closed circles) and 70% HFIP (open and closed triangles). The presence of 70% TFA completely abolished fluorescamine reaction (open and closed squares). Lines are linear fits to the data. (*b*) Concentration of Aβ42 determined using the fluorescamine method in the absence and presence of 70% DMSO or 70% HFIP. Error bars are the standard deviations of three independent measurements.

Preparation of Aβ oligomers, such as ADDLs [29], often involves solubilizing Aβ in DMSO. Owing to its high absorbance at 280 nm, the presence of DMSO may not allow for reliable concentration measurements using UV absorbance. Here we investigated whether DMSO is compatible with the fluorescamine method. Figure 3*a* shows the standard curve in the presence of 70% DMSO. The linear relationship remains, with an *r*^2^ value at greater than 0.99, but the slope with 70% DMSO is dramatically lower than the slope without DMSO. Additional fluorescamine measurements with lower concentrations of DMSO show that the slope of the standard curve increases with decreasing concentrations of DMSO (data not shown). Therefore, it is important that, for accurate concentration measurements, the standard curves should be obtained according to the buffer compositions of the specific experiments.

Similar to DMSO, hexafluoroisopropanol (HFIP) and trifluoroacetic acid (TFA) are two organic solvents that are commonly used to solubilize and disaggregate Aβ. Here we also examined if the fluorescamine method is compatible with HFIP and TFA. We found that a linear relationship exists between fluorescamine fluorescence and lysozyme concentration in the presence of 70% HFIP (figure 3*a*). However, the presence of 70% TFA completely abolished the fluorescamine reaction (figure 3*a*).

Consequent concentration determination of the Aβ42 sample demonstrates good agreement between the measurements in the absence of any organic solvents and in the presence of 70% DMSO or 70% HFIP (figure 3*b*). However, the slope of the standard curve is lower with 70% DMSO or 70% HFIP, indicating that sensitivity may be lower. DMSO also gives a higher background signal in the absence of proteins. Because HFIP is a volatile solvent, extra care must be taken when performing assays with HFIP, which evaporates quickly and may contribute to larger errors in concentration measurements (figure 3*b*).

### 2.4. Optimal fluorescamine concentrations for maximal sensitivity

**Figure 4.**
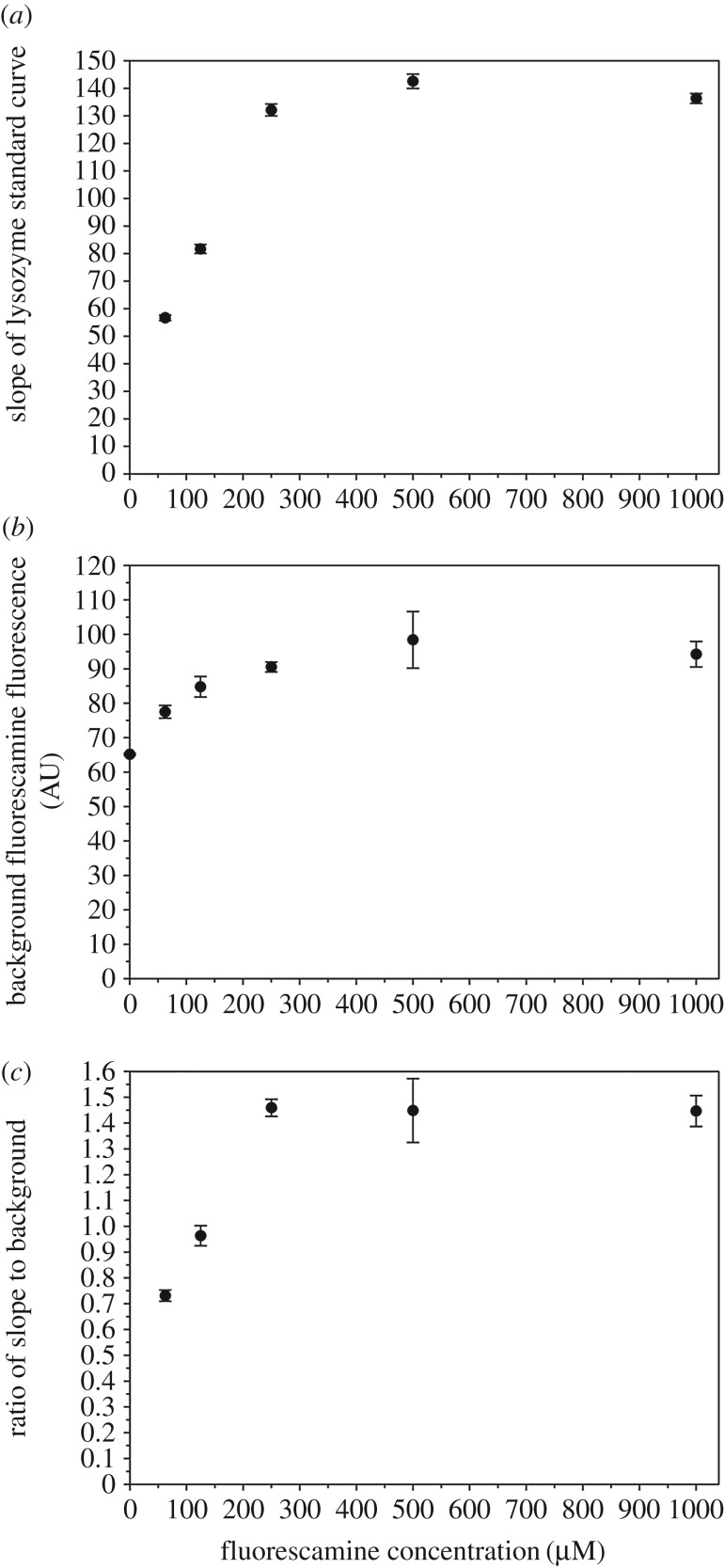
Optimal fluorescamine concentrations for best sensitivity. (*a*) The slope of the lysozyme standard curve from figure 1 as a function of fluorescamine concentrations used. (*b*) Background fluorescamine fluorescence in the absence of proteins from figure 1 as a function of fluorescamine concentration used. (*c*) Slope to background ratio as a function of fluorescamine concentration used.

We assessed the effect of fluorescamine concentration on the sensitivity of protein quantification using the standard curves. Such sensitivity is related to both the slope of the standard curve and the background fluorescence. A steeper standard curve (with a larger slope) corresponds to higher sensitivity toward changes in protein concentration. As shown in figure 4*a*, the slope of the standard curve increases with increasing fluorescamine concentrations from 62.5 µM to 250 µM, and approaches plateau above 250 µM. Background noise must also be considered a factor in analysing sensitivity, because fluorescamine gives a low but evident fluorescence signal in the absence of proteins. Because of this, background noise can take up a significant portion of overall fluorescence intensity when protein concentration is low. As shown in figure 4*b*, the background noise increases with increasing concentrations of fluorescamine from 62.5 µM to 250 µM, approaching plateau above 250 µM. Therefore, we use a ratio of slope to background noise as a measure of sensitivity, as shown in figure 4*c*. Maximum sensitivity is reached at 250 µM fluorescamine and plateaus thereafter.

**Figure 5.**
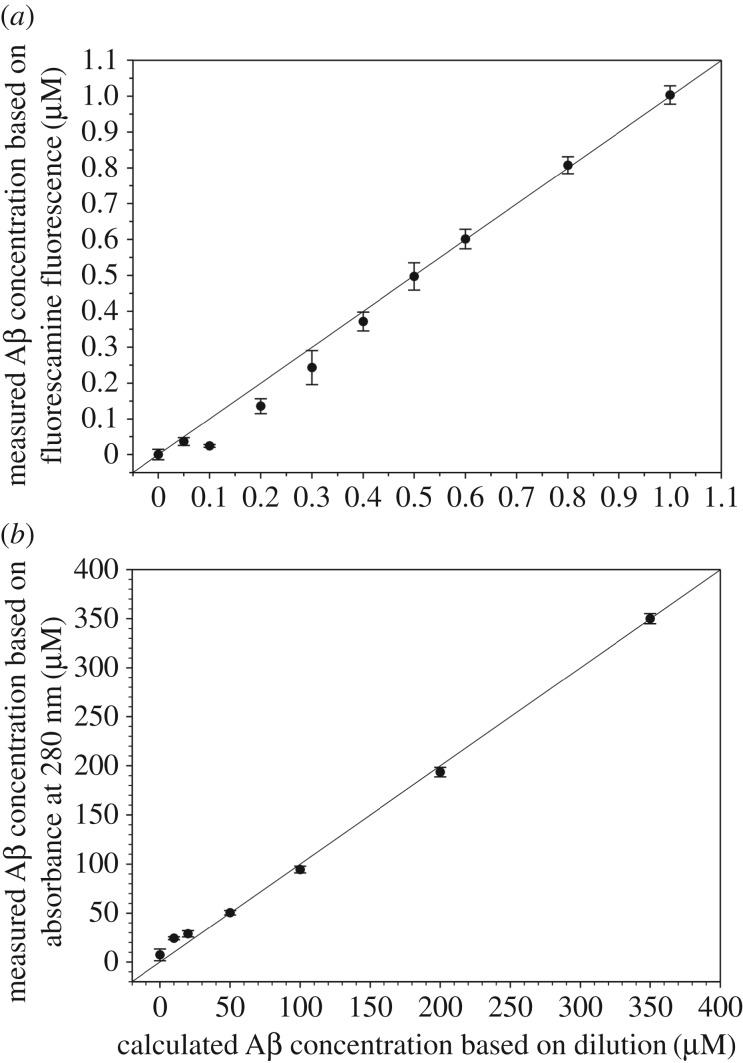
Comparison of detection sensitivity for the fluorescamine and absorbance methods. (*a*) Measured Aβ42 concentrations using 500 µM fluorescamine are plotted as a function of calculated concentrations based on dilution. A data point on the diagonal line means perfect agreement between measured and calculated concentrations. Deviation from the diagonal line suggests that the measurements are losing accuracy. The measured concentrations for 0.5 µM or higher agree perfectly with calculated concentrations. The measurements for the 0.4 µM, 0.3 µM and 0.2 µM are 7%, 19% and 32% off from the calculated concentrations, respectively. Concentrations at 0.1 µM or lower gave fluorescence signal similar to background. (*b*) Measured Aβ42 concentrations using absorbance at 280 nm are plotted as a function of calculated concentrations based on dilution. Perfect agreements are shown at Aβ concentrations of 50 µM or higher. Deviations of 44% and 140% from calculated values are found for 20 and 10 µM Aβ. With perfect agreements at 0.5 µM Aβ for the fluorescamine method and at 50 µM Aβ for the absorbance method, the fluorescamine method is 100 times more sensitive than the absorbance method.

We also compared the Aβ detection sensitivity of the fluorescamine method and absorbance method (figure 5). For the fluorescamine method, we started from an Aβ42 sample in PBS at 1 µM concentration, which was determined using fluorescamine. Then this Aβ42 sample was diluted to 0.8, 0.6, 0.5 0.4, 0.3, 0.2, 0.1 and 0.05 µM, and the concentration was determined using fluorescamine. As shown in figure 5*a*, we found perfect agreement between measured concentrations and calculated concentrations when Aβ concentration is 0.5 µM or higher. Aβ concentrations at 0.4, 0.3 and 0.2 µM showed a difference of 7%, 19% and 32% between measured and calculated values. At concentrations of 0.1 µM or lower, the signal is not significantly higher than background.

For the absorbance method, we started from an Aβ42 sample dissolved in a high pH denaturing buffer containing 20 mM CAPS (pH 11) and 7 M GdnHCl at a concentration of 350 µM, which was determined using absorbance at 280 nm. We then diluted this Aβ sample to 200, 100, 50, 20 and 10 µM, and the concentration was determined again using absorbance. As shown in figure 5*b*, we found perfect agreement when Aβ concentration is 50 µM or higher. However, a deviation of 44% from the calculated value was found for the 20 µM Aβ42 sample, and a deviation of greater than 100% from the calculated value was found for the 10 µM Aβ42 sample, suggesting that absorbance at 280 nm is not suitable for Aβ concentrations at 20 µM or lower. At 20 µM, the theoretical absorbance value of Aβ is approximately 0.023, a value that is generally considered too low for accurate measurement of absorbance.

### 2.5. Evaluation of fluorescamine method using aggregation kinetics

Eventually, the real test of the rigour of a quantification method is how it performs in experimental applications. Most *in vitro* Aβ aggregation experiments involve the preparation of oligomers and fibrils, or the study of aggregation kinetics. Here we use aggregation kinetics to evaluate the accuracy of the fluorescamine method in determining Aβ concentration.

**Figure 6.**
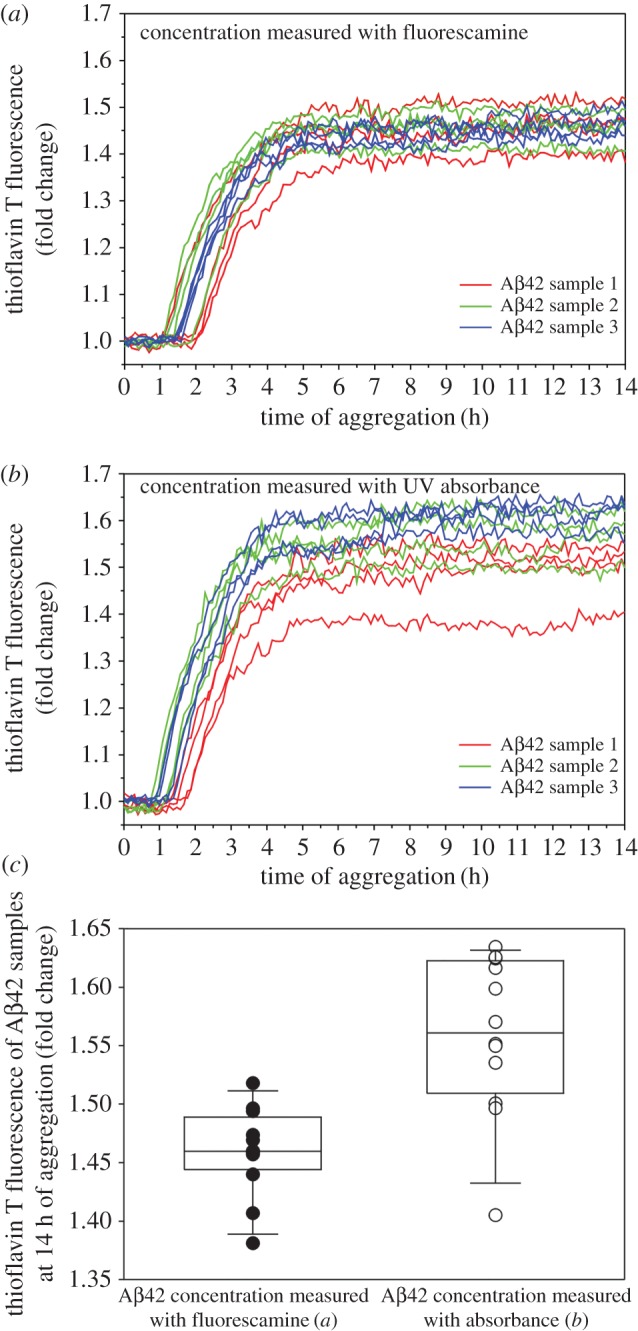
Comparison of Aβ42 aggregation kinetics with concentrations determined using fluorescamine (*a*) or absorbance (*b*). (*c*) Thioflavin T fluorescence at aggregation plateau for all Aβ42 samples in (*a*) and (*b*) are superimposed on box plots to show the spread of fluorescence intensity. Whiskers of the box plot indicate the 10th and 90th percentiles. Three Aβ42 samples were prepared separately at approximately 30–40 µM in PBS buffer. For each sample, the concentration was determined using both fluorescamine and absorbance. Then four repeats of aggregation experiments were set up for each sample at 5 μM. The overall fluorescence intensity reaches similar values at aggregation plateau when concentrations are determined using fluorescamine (*a*) and absorbance (*b*), suggesting a general agreement of the two methods. However, aggregation curves for samples using the fluorescamine method are more tightly clustered together (*c*), suggesting better consistency with the fluorescamine method.

We prepared three independent Aβ42 samples in PBS buffer at concentrations of 30–40 µM, as determined by absorbance at 280 nm. Aβ42 concentrations at this range ensure relatively accurate concentration measurements using absorbance. The concentration of each sample was also determined using fluorescamine. Then we set up aggregation kinetics experiments at 5 µM concentration, with four repeats for each sample. The results, as shown in figure 6, show that similar aggregation kinetics curves were obtained with concentrations determined with fluorescamine (figure 6*a*) and absorbance (figure 6*b*). The two sets of experiments have similar lag time and similar fluorescence intensity at aggregation plateau, which is a quantitative measure of the amount of amyloid fibrils [3]. Therefore, these results show a general agreement between the fluorescamine and absorbance methods. However, the kinetics curves with the fluorescamine method are more tightly clustered together than those with the absorbance method (figure 6*c*), suggesting that the fluorescamine method has better consistency for low Aβ concentrations.

**Figure 7.**
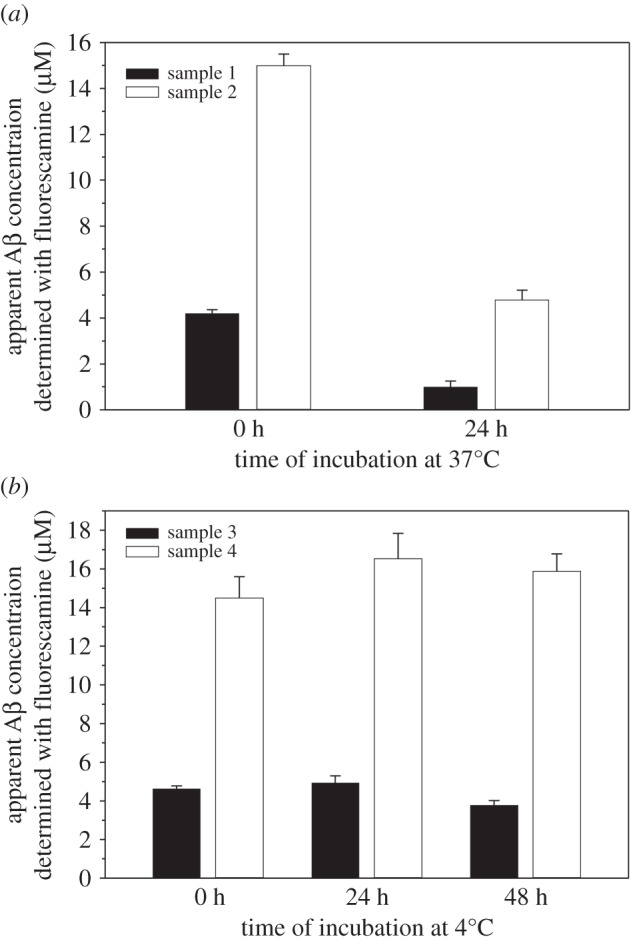
Concentration measurements with fluorescamine for Aβ samples incubated at 37°C (*a*) or 4°C (*b*). Four Aβ42 samples (referred to as 1, 2, 3 and 4) were prepared separately in PBS buffer. Incubation at 37°C leads to rapid aggregation of Aβ42, and consequent reduction in apparent concentrations measured using the fluorescamine method (*a*). This is due to the reduction of the number of solvent-accessible lysine side chains as a result of protein aggregation. By contrast, only small changes were observed for Aβ42 samples incubated at 4°C at similar concentrations (*b*), suggesting that Aβ42 remains soluble at 4°C during the incubation period.

We next investigated whether fluorescamine can be used to monitor the Aβ concentration during the process of aggregation. If lysine residues were exposed in the fibrils, we would be able to determine Aβ concentration for not just soluble Aβ, but also aggregated Aβ. As shown in figure 7*a*, upon incubation at 37°C for 24 h, the apparent Aβ42 concentration as determined using fluorescamine fluorescence was reduced by approximately 70%, suggesting that the majority of the lysine residues became buried in the fibrils. By contrast, incubation of Aβ42 samples at 4°C results in only small changes in apparent concentration for at least 48 h (figure 7*b*), suggesting that Aβ42 samples remain soluble at 4°C during the incubation period.

## 3. Conclusion

Absorbance at 280 nm has proven to be a fast and reliable way to quantify proteins, but its sensitivity is low. In Aβ aggregation experiments, especially for Aβ42, protein concentration must be kept in the low micromolar range to prevent rapid fibrillization, and more sensitive protein quantification methods are required. Here we present a fluorescamine method for quantification of Aβ *in vitro.* We show that fluorescamine fluorescence correlates linearly with lysozyme concentration with sub-micromolar sensitivity. Concentration measurements with fluorescamine can be performed in the presence of DMSO, making fluorescamine an ideal reagent for Aβ oligomer preparations where DMSO is often a required solvent. Using fluorescamine, highly consistent aggregation kinetics can be obtained. To facilitate reproducibility in Aβ studies, we recommend the fluorescamine method to be used for routine and on-the-fly Aβ quantification for a wide range of Aβ experimental applications.

## 4. Material and methods

### 4.1. Preparation of Aβ proteins

Aβ was prepared as previously described [3,4,30]. Briefly, Aβ protein was expressed in *Escherichia coli* as a fusion protein, GroES-ubiquitin-Aβ. After purification, the fusion protein was cleaved to obtain full-length Aβ without any extra residues. Finally, Aβ was lyophilized and stored at −80°C.

### 4.2. Standard curves for the fluorescamine method

A stock solution of 50 mM fluorescamine (Acros Organics, pure grade) was made by adding acetonitrile to the glass bottle shipped from the manufacturer. The concentration of fluorescamine was calculated based on the weight (supplied by the manufacturer). Other concentrations of fluorescamine stock were then diluted using acetonitrile. A stock solution of 50 µM hen egg white lysozyme (Fisher Bioreagents, crystalline powder) was made in deionized water, and the concentration was determined using absorbance at 280 nm and an extinction coefficient of 38 000 M^−1^ cm^−1^ [31]. Other concentrations of lysozyme stock were then diluted using deionized water. Solutions of BSA were diluted from 2 mg ml^−1^ Albumin Standard Ampules (Thermo Scientific Pierce) using deionized water.

Standard curves were prepared using stock concentrations of hen egg white lysozyme and fluorescamine. First, 5 µl of lysozyme stock solutions at concentrations of 1, 2, 4, 6, 8, 10, 12, 15 and 20 µM were mixed with 40 µl of PBS buffer (50 mM phosphate, 140 mM NaCl, pH 7.4). The blank is a mixture of 5 µl of deionized water and 40 µl of PBS buffer. Second, 5 µl of fluorescamine stock solution at concentrations of 0.625, 1.25, 2.5, 5 and 10 mM were pipetted in and immediately mixed by stirring with the pipetting tip. We found that vortexing gave higher background noise readings and should be avoided. Amine concentrations were calculated by multiplying lysozyme concentrations by 7 (as each lysozyme protein contains seven primary amine groups). Standard curves with BSA were also prepared using 500 µM fluorescamine and the concentration of BSA was converted to amine concentration by multiplying by 60 (as each BSA contains 60 primary amine groups).

Standard curves with 70% DMSO, 70% HFIP or 70% TFA were prepared similarly as the above except 35 µl of DMSO, HFIP or TFA were used to substitute 35 µl of PBS buffer.

After addition of fluorescamine, all samples were immediately transferred to a black 384-well Nonbinding Surface microplate with clear bottom (Corning product no. 3655). Fluorescence readings were measured at room temperature using a Spectramax Gemini EM plate reader (Molecular Devices), with excitation at 390 nm and emission at 478 nm. Least-squares regression analyses on the standard plots were performed using SigmaPlot (Systat Software, Inc).

### 4.3. Concentration measurements of Aβ40 using fluorescamine and absorbance

One tube of Aβ40 powder (approx. 200 µg) was dissolved in 500 µl of HFIP, split into two tubes, and dried with a gentle stream of argon gas. For the absorbance method, one tube of Aβ40 was then dissolved in CG buffer (20 mM CAPS, 7 M GdnHCl, pH 11). The volume of CG buffer was chosen so that the absorbance reading would be between 0.5 and 1.0. The sample was used for absorbance measurement without further dilution. Absorbance at 280 nm was measured using a quartz microcuvette with 1 cm path length on a Jasco V-630 spectrophotometer. Concentration was calculated using an extinction coefficient of 1280 M^−1^ cm^−1^ [5]. The other tube of Aβ40 was dissolved in one volume of 10 mM NaOH. Then eight volumes of PBS were added, followed by one volume of 10 mM HCl for neutralization. To measure concentration using fluorescamine, 5 µl of Aβ40 sample was mixed with 40 µl of PBS and 5 µl of fluorescamine stock solutions. The sample was further diluted if needed so that fluorescence intensity readings would fall in the middle of the standard curves. For the fluorescamine method with DMSO or HFIP, 35 µl of DMSO or HFIP were used to substitute 35 µl of PBS buffer. Fluorescence measurements were performed the same way as the standard curves. Concentration was determined using the five different fluorescamine concentrations, with the standard curves that were established above.

### 4.4. Aβ42 fibrillization kinetics

Three tubes of HFIP-treated Aβ42 powder were dissolved in 1 ml of CG buffer for each tube, and buffer-exchanged to PBS using a 5 ml HiTrap desalting column (GE healthcare). The concentration of Aβ42 was determined using both absorbance and fluorescamine. Aβ42 was then diluted to 5 µM in PBS buffer containing 20 µM thioflavin T, and 50 µl of aggregation sample was transferred to a black 384-well Nonbinding Surface microplate with clear bottom (Corning product no. 3655), and sealed with a polyester-based sealing film (Corning product no. PCR-SP). Four repeats of aggregation experiments were performed for each sample. The fluorescence was measured through the bottom of the microplate with an excitation filter of 450 nm and an emission filter of 490 nm using a Victor 3 V plate reader (Perkin Elmer). The aggregation was started by putting the microplate in the reader and starting incubation at 37°C without agitation.

## Data accessibility

Data available from the Dryad Digital Repository: http://dx.doi.org/10.5061/dryad.7mg3h [32].

## References

[1] Thal DR, Walter J, Saido TC, Fändrich M 2015 Neuropathology and biochemistry of Aβ and its aggregates in Alzheimer's disease. Acta Neuropathol. 129, 167–182. (doi:10.1007/s00401-014-1375-y) 25534025 10.1007/s00401-014-1375-y

[2] Selkoe DJ, Hardy J 2016 The amyloid hypothesis of Alzheimer's disease at 25 years. EMBO Mol. Med. 8, 595–608. (doi:10.15252/emmm.201606210) 27025652 10.15252/emmm.201606210 PMC4888851

[3] Xue C, Lin TY, Chang D, Guo Z 2017 Thioflavin T as an amyloid dye: fibril quantification, optimal concentration and effect on aggregation. R. Soc. open sci. 4, 160696 (doi:10.1098/rsos.160696) 28280572 10.1098/rsos.160696 PMC5319338

[4] Tran J, Chang D, Hsu F, Wang H, Guo Z 2017 Cross-seeding between Aβ40 and Aβ42 in Alzheimer's disease. FEBS Lett. 591, 177–185. (doi:10.1002/1873-3468.12526) 27981583 10.1002/1873-3468.12526 PMC5235972

[5] Edelhoch H 1967 Spectroscopic determination of tryptophan and tyrosine in proteins. Biochemistry 6, 1948–1954. (doi:10.1021/bi00859a010) 6049437 10.1021/bi00859a010

[6] Elbassal EA, Liu H, Morris C, Wojcikiewicz EP, Du D 2016 Effects of charged cholesterol derivatives on Aβ40 amyloid formation. J. Phys. Chem. B 120, 59–68. (doi:10.1021/acs.jpcb.5b09557) 26652010 10.1021/acs.jpcb.5b09557 PMC4959543

[7] Pauwels K et al. 2012 Structural basis for increased toxicity of pathological Aβ42:Aβ40 ratios in Alzheimer disease. J. Biol. Chem. 287, 5650–5660. (doi:10.1074/jbc.M111.264473) 22157754 10.1074/jbc.M111.264473 PMC3285338

[8] Chromy BA et al. 2003 Self-assembly of Aβ1-42 into globular neurotoxins. Biochemistry 42, 12 749–12 760. (doi:10.1021/bi030029q) 10.1021/bi030029q 14596589

[9] Balducci C et al. 2010 Synthetic amyloid-β oligomers impair long-term memory independently of cellular prion protein. Proc. Natl Acad. Sci. USA 107, 2295–2300. (doi:10.1073/pnas.0911829107) 20133875 10.1073/pnas.0911829107 PMC2836680

[10] Laurén J, Gimbel DA, Nygaard HB, Gilbert JW, Strittmatter SM 2009 Cellular prion protein mediates impairment of synaptic plasticity by amyloid-β oligomers. Nature 457, 1128–1132. (doi:10.1038/nature07761) 19242475 10.1038/nature07761 PMC2748841

[11] Freir DB et al. 2011 Interaction between prion protein and toxic amyloid β assemblies can be therapeutically targeted at multiple sites. Nat. Commun. 2, 336 (doi:10.1038/ncomms1341) 21654636 10.1038/ncomms1341 PMC3156817

[12] Sinnen BL, Bowen AB, Gibson ES, Kennedy MJ 2016 Local and use-dependent effects of β-amyloid oligomers on NMDA receptor function revealed by optical quantal analysis. J. Neurosci. 36, 11 532–11 543. (doi:10.1523/JNEUROSCI.1603-16.2016) 10.1523/JNEUROSCI.1603-16.2016 PMC5125218 27911757

[13] Lacor PN et al. 2004 Synaptic targeting by Alzheimer's-related amyloid beta oligomers. J. Neurosci. 24, 10 191–10 200. (doi:10.1523/JNEUROSCI.3432-04.2004) 10.1523/JNEUROSCI.3432-04.2004 PMC6730194 15537891

[14] Udenfriend S, Stein S, Böhlen P, Dairman W, Leimgruber W, Weigele M 1972 Fluorescamine: a reagent for assay of amino acids, peptides, proteins, and primary amines in the picomole range. Science 178, 871–872. (doi:10.1126/science.178.4063.871) 5085985 10.1126/science.178.4063.871

[15] Stein S, Böhlen P, Udenfriend S 1974 Studies on the kinetics of reaction and hydrolysis of fluorescamine. Arch. Biochem. Biophys. 163, 400–403. (doi:10.1016/0003-9861(74)90491-3) 4859504 10.1016/0003-9861(74)90491-3

[16] De Bernardo S, Weigele M, Toome V, Manhart K, Leimgruber W, Böhlen P, Stein S, Udenfriend S 1974 Studies on the reaction of fluorescamine with primary amines. Arch. Biochem. Biophys. 163, 390–399. (doi:10.1016/0003-9861(74)90490-1) 4859505 10.1016/0003-9861(74)90490-1

[17] Klein B, Standaert F 1976 Fluorometry of plasma amino nitrogen, with use of fluorescamine. Clin. Chem. 22, 413–416. 1253422

[18] Castell JV, Cervera M, Marco R 1979 A convenient micromethod for the assay of primary amines and proteins with fluorescamine. A reexamination of the conditions of reaction. Anal. Biochem. 99, 379–391. (doi:10.1016/S0003-2697(79)80022-6) 42326 10.1016/s0003-2697(79)80022-6

[19] Bode J 1979 On the reactions of fluorescamine with chromosomal proteins. Anal. Biochem. 99, 274–280. (doi:10.1016/S0003-2697(79)80006-8) 517740 10.1016/s0003-2697(79)80006-8

[20] Lorenzen A, Kennedy SW 1993 A fluorescence-based protein assay for use with a microplate reader. Anal. Biochem. 214, 346–348. (doi:10.1006/abio.1993.1504) 8250247 10.1006/abio.1993.1504

[21] Lowry OH, Rosebrough NJ, Farr AL, Randall RJ 1951 Protein measurement with the Folin phenol reagent. J. Biol. Chem. 193, 265–275. 14907713

[22] Bradford MM 1976 A rapid and sensitive method for the quantitation of microgram quantities of protein utilizing the principle of protein-dye binding. Anal. Biochem. 72, 248–254. (doi:10.1016/0003-2697(76)90527-3) 942051 10.1016/0003-2697(76)90527-3

[23] Smith PK et al. 1985 Measurement of protein using bicinchoninic acid. Anal. Biochem. 150, 76–85. (doi:10.1016/0003-2697(85)90442-7) 3843705 10.1016/0003-2697(85)90442-7

[24] Morshedi D, Ebrahim-Habibi A, Moosavi-Movahedi AA, Nemat-Gorgani M 2010 Chemical modification of lysine residues in lysozyme may dramatically influence its amyloid fibrillation. Biochim. Biophys. Acta 1804, 714–722. (doi:10.1016/j.bbapap.2009.11.012) 19945549 10.1016/j.bbapap.2009.11.012

[25] Shih P, Holland DR, Kirsch JF 1995 Thermal stability determinants of chicken egg-white lysozyme core mutants: hydrophobicity, packing volume, and conserved buried water molecules. Protein Sci. 4, 2050–2062. (doi:10.1002/pro.5560041010) 8535241 10.1002/pro.5560041010 PMC2142977

[26] Mir MM, Fazili KM, Abul Qasim M 1992 Chemical modification of buried lysine residues of bovine serum albumin and its influence on protein conformation and bilirubin binding. Biochim. Biophys. Acta 1119, 261–267. (doi:10.1016/0167-4838(92)90212-V) 1547271 10.1016/0167-4838(92)90212-v

[27] Noble JE, Knight AE, Reason AJ, Matola AD, Bailey MJA 2007 A comparison of protein quantitation assays for biopharmaceutical applications. Mol. Biotechnol. 37, 99–111. (doi:10.1007/s12033-007-0038-9) 17914170 10.1007/s12033-007-0038-9

[28] Bantan-Polak T, Kassai M, Grant KB 2001 A comparison of fluorescamine and naphthalene-2,3-dicarboxaldehyde fluorogenic reagents for microplate-based detection of amino acids. Anal. Biochem. 297, 128–136. (doi:10.1006/abio.2001.5338) 11673879 10.1006/abio.2001.5338

[29] Lambert MP et al. 2001 Vaccination with soluble Aβ oligomers generates toxicity-neutralizing antibodies. J. Neurochem. 79, 595–605. (doi:10.1046/j.1471-4159.2001.00592.x) 11701763 10.1046/j.1471-4159.2001.00592.x

[30] Gu L, Liu C, Guo Z 2013 Structural insights into Aβ42 oligomers using site-directed spin labeling. J. Biol. Chem. 288, 18 673–18 683. (doi:10.1074/jbc.M113.457739) 10.1074/jbc.M113.457739 PMC3696641 23687299

[31] Gill SC, von Hippel PH 1989 Calculation of protein extinction coefficients from amino acid sequence data. Anal. Biochem. 182, 319–326. (doi:10.1016/0003-2697(89)90602-7) 2610349 10.1016/0003-2697(89)90602-7

[32] Xue C, Lee YK, Tran J, Chang D, Guo Z 2017 Data from: A mix-and-click method to measure amyloid-β concentration with sub-micromolar sensitivity. Dryad Digital Repository. (http://dx.doi.org/10.5061/dryad.7mg3h) 10.1098/rsos.170325 PMC5579099 28878984

